# Ultra-fast biparametric MRI in prostate cancer assessment: Diagnostic performance and image quality compared to conventional multiparametric MRI

**DOI:** 10.1016/j.ejro.2025.100635

**Published:** 2025-01-21

**Authors:** Antonia M. Pausch, Vivien Filleböck, Clara Elsner, Niels J. Rupp, Daniel Eberli, Andreas M. Hötker

**Affiliations:** a Diagnostic and Interventional Radiology, University Hospital Zurich, Switzerland; b Department of Pathology and Molecular Pathology, University Hospital Zurich, Switzerland; c Faculty of Medicine, University of Zurich, Switzerland; d Department of Urology, University Hospital Zurich, Switzerland

**Keywords:** Prostate cancer, Deep learning, Ultra-fast bpMRI, MpMRI, PI-RADS

## Abstract

**Purpose:**

To compare the diagnostic performance and image quality of a deep-learning-assisted ultra-fast biparametric MRI (bpMRI) with the conventional multiparametric MRI (mpMRI) for the diagnosis of clinically significant prostate cancer (csPCa).

**Methods:**

This prospective single-center study enrolled 123 biopsy-naïve patients undergoing conventional mpMRI and additionally ultra-fast bpMRI at 3 T between 06/2023–02/2024. Two radiologists (R1: 4 years and R2: 3 years of experience) independently assigned PI-RADS scores (PI-RADS v2.1) and assessed image quality (mPI-QUAL score) in two blinded study readouts. Weighted Cohen’s Kappa (κ) was calculated to evaluate inter-reader agreement. Diagnostic performance was analyzed using clinical data and histopathological results from clinically indicated biopsies.

**Results:**

Inter-reader agreement was good for both mpMRI (κ = 0.83) and ultra-fast bpMRI (κ = 0.87). Both readers demonstrated high sensitivity (≥94 %/≥91 %, R1/R2) and NPV (≥96 %/≥95 %) for csPCa detection using both protocols. The more experienced reader mostly showed notably higher specificity (≥77 %/≥53 %), PPV (≥62 %/≥45 %), and diagnostic accuracy (≥82 %/≥65 %) compared to the less experienced reader. There was no significant difference in the diagnostic performance of correctly identifying csPCa between both protocols (p > 0.05). The ultra-fast bpMRI protocol had significantly better image quality ratings (p < 0.001) and achieved a reduction in scan time of 80 % compared to conventional mpMRI.

**Conclusion:**

Deep-learning-assisted ultra-fast bpMRI protocols offer a promising alternative to conventional mpMRI for diagnosing csPCa in biopsy-naïve patients with comparable inter-reader agreement and diagnostic performance at superior image quality. However, reader experience remains essential for diagnostic performance.

## Highlights

• DL-assisted ultra-fast bpMRI and mpMRI with comparable performance in csPCa detection.
• Superior image quality of DL-assisted ultra-fast bpMRI.
• Enhancing PCa management by offering a quicker, patient-friendly imaging option.

## 1 Introduction

In 2020, approximately 1.4 million men were newly diagnosed with prostate cancer (PCa), a number projected to reach almost 2.3 million by 2040 [1]. This increase is to a large degree attributed to population growth, an aging demographic, and the widespread use of prostate-specific antigen (PSA) testing [1]. As a result, healthcare expenses are anticipated to rise, putting additional strain on the healthcare system. This situation may also restrict the accessibility to magnetic resonance imaging (MRI) in certain areas and countries, which has become an integral part of prostate cancer assessment [2], [3].

The current PI-RADS v2.1 guideline [4] provides detailed technical instructions for performing prostate MRI, typically requiring about 30 minutes per scan, influenced by prostate size and scanner specifications. Consequently, there is an attempt to refine and expedite this process. A key aspect of the optimization strategy involves accelerating and prioritizing the "core" sequences (T2-weighted and diffusion-weighted sequences), while removing sequences considered to be less important, i.e., contrast enhanced-sequences, from the complete multiparametric MRI (mpMRI) protocol [5].

Recently, innovations in MR techniques based on machine learning methods have been introduced, which have the potential to significantly reduce image noise, enhance signal-to-noise ratio, and optimize image sharpness, consequently reducing acquisition time without compromising image quality [6], [7], [8], [9]. By using these new techniques and focusing on the “core” sequences which are essential to diagnose prostate cancer on MRI, an ultra-fast biparametric MRI (bpMRI) protocol with a scan time of < 5 min is achievable.

With growing demands on global healthcare and limited resources, finding efficient, cost-effective diagnostic and treatment methods for prostate cancer is crucial. Due to reduced scanning times and eliminating the use of contrast media, accelerated protocols could improve patient comfort, lower healthcare costs and extend access to prostate MRI to more men, including those unable to endure long scan times due to claustrophobia or back pain [10].

The purpose of this study is therefore to compare the diagnostic performance and image quality of an ultra-fast bpMRI to the conventional full mpMRI of the prostate.

## 2 Methods

### 2.1 Study design

This prospective single-center study (ClinicalTrials number: NCT05903781), approved by the institutional review board (*blinded for review*), enrolled consecutive biopsy-naïve patients aged 18 years or older from June 2023 to February 2024, who met the clinical indications for a prostate MRI according to established guidelines (specifically, the EAU guideline [11]), due to raised PSA levels, and/or abnormal digital rectal examinations, and/or suspicious transrectal ultrasound (TRUS). Written informed consent was obtained from each study participant prior to MRI. The final study cohort included only patients without a previous history of prostate cancer, who underwent complete MRI examinations, including study sequences, and post-MRI clinical reassessment. Patients who did not meet these inclusion criteria were excluded.

### 2.2 MRI protocol and image reconstruction

The MR scans were performed on 3.0 Tesla MR scanners (MAGNETOM Vida fit, Siemens Healthineers, Erlangen, Germany). All patients underwent a conventional clinical mpMRI, with additional sequences incorporated as part of the study's ultra-fast bpMRI protocol. In accordance with current PI-RADS v2.1 guidelines [4], the mpMRI comprises T2-weighted (T2w) TSE sequences in three planes, diffusion-weighted imaging (DWI, b-values of 100, 600 and 1000 s/mm^2^, calculated b-value of 2000 s/mm^2^) and dynamic contrast-enhanced (DCE) MRI with injection of gadoterate meglumine (Dotarem, Guerbet, Villepinte, France) as a contrast agent (dose of 0.1 mmol/kg body weight).

The ultra-fast bpMRI study protocol included axial T2w TSE sequences and DWI (b-values of 50 and 800 s/mm^2^, calculated b-value of 1400 s/mm^2^), which were acquired additionally prior to contrast medium application. The T2w study sequence was generated using a MRI reconstruction feature which is part of the MRI scanner’s reconstruction pipeline (“Deep resolve”, Siemens Healthineers, Erlangen, Germany) [12]. The technology uses the original raw k-space data in combination with precalculated coil sensitivity maps. This serves as input for an iterative process and deep learning (DL)-algorithms, specifically variational and convolutional neural networks, in the MRI reconstruction process. It employs an iterative process where a deep neural network alternates with data-consistency updates, combining physical MRI models with data-driven models for high performance. Consequently, the reconstruction framework consists of multiple cascades, each with a data consistency step and convolutional neural network-based regularization to minimize noise from acceleration [8], [12], [13]. DL-assisted image reconstruction can therefore simultaneously overcome all three major limitations of MR imaging — image resolution, signal-to-noise ratio and acquisition speed — while also being applicable to 2D Cartesian sampling [12]. The study sequences archive acquisition time savings of approximately 80 % (acquisition times: 3 min 28 s for full ultra-fast bpMRI vs. 20 mins 33 s for full standard mpMRI). Table 1 provides a detailed overview of the acquisition parameters of the T2w und DWI sequences for both protocols.

**Table 1:** Typical Imaging parameters of the T2-weighted and DWI sequences of both protocols.

	**mpMRI**	**ultra-fast bpMRI**
**Acquisition time (full protocol)**	20 min 33 s	3 min 28 s (approx. - 80 %)
**Sequence**	**T2 TSE**	**T2 TSE**
**Acquisition time**	3 min 12 s	1 min 56 s (approx. - 40 %)
**Orientation**	axial	axial
**Number of slices**	27	27
**Slice thickness (mm)**	3	3
**Gap**	0 %	0 %
**Field of view (mm**^**2**^**)**	180 × 180	179 × 179
**Matrix size**	704 × 704	640 × 640
**Pixel spacing (mm)**	0.26 × 0.26	0.28 × 0.28
**PAT factor**	3	4
**Number of excitations**	3	2
**TR (ms)**	4100	4030
**TE (ms)**	102	101
**Flip Angle (º)**	140	140
**Echo train length**	25	25
**Sequence**	**DWI**	**DWI**
**Sequence type**	EPI	EPI
**Acquisition time**	4 min 50 s	1 min 32 s (approx. - 70 %)
**Orientation**	axial	axial
**Number of slices**	27	27
**Slice thickness (mm)**	3	3
**Field of view (mm**^**2**^**)**	101 × 199	101 × 199
**Matrix size**	116 × 228	116 × 224
**Pixel spacing (mm)**	0.88 × 0.88	0.88 × 0.88
**Number of excitations**	2/3/10	2/3
**B-values (s/mm**^**2**^**)**	100, 600, 1000, calculated 2000	50, 800, calculated 1400
**TR (ms)**	4800	4500
**TE (ms)**	70	68
**Flip Angle (º)**	90	90
**Echo train length**	61	61

### 2.3 MRI analysis

All conventional clinical mpMRIs were reviewed by board-certified radiologists as part of the clinical routine on a Picture Archiving and Communication System (PACS) workstation and assigned a PI-RADS v2.1 score and this score informed clinical-decision making (i.e., decision to perform biopsy). Additionally, for the purpose of this study, two radiologists — one with 4 years (Reader 1, 300 prostate MRIs/year) and the other with 3 years (Reader 2, 150 prostate MRIs/year) of experience in reading prostate MRIs — independently reviewed the conventional mpMRI and ultra-fast bpMRI. Both readers were blinded to all clinical or histopathological details, except for the knowledge that the MRI was conducted for prostate cancer detection. In the first readout, the two readers evaluated the mpMRIs. During the second readout, the readers evaluated the ultra-fast bpMRI images at least 4 weeks after the initial readout to prevent recall bias. They assessed the images independently and were blinded to their prior mpMRI evaluations. The PI-RADS score for one index lesion was documented in accordance with the current PIRADS v2.1 guideline [4] during both readouts for each patient. In cases where multiple lesions were present, the lesion with the highest PIRADS score was designated as the index lesion. If several lesions had the same highest PIRADS score, the lesion with the biggest size in mm or extraprostatic extension was defined the index lesion. The designated index lesion for every patient was noted based on four prostate quadrants (anterior right/left, posterior right/left), so that it was ensured that the statistical analysis was based on both readers evaluating identical lesions.

Image quality of the mpMRIs and ultra-fast bpMRIs was assessed by both readers using a 4-point Likert scale in accordance with the modified PI-QUAL (mPI-QUAL) score, as it also allows for the assessment of bpMRI without DCE sequences [14]. A score of 1 indicates non-diagnostic image quality, while a score of 4 indicates optimal diagnostic quality for two or more mpMRI sequences [14], [15].

### 2.4 Post-MRI clinical reassessment and standard-of-reference

After the clinical mpMRI, each patient underwent clinical reassessment within the Department of Urology to determine the further course of action and evaluate the necessity of any additional workup. This clinical reassessment was not part of our prospective study protocol and was therefore only based on the results of the clinical mpMRI report. The indication for prostate biopsy was based on EAU guidelines [11], which involved suspicious lesions on the clinical mpMRI (PI-RADS score ≥ 3) in correlation with PSA-density assessment. Accordingly, board-certified urologists from our hospital performed software-based MRI/TRUS fusion targeted biopsies, in conjunction with systematic biopsies, as per standard clinical practice. In instances where the MRI examination did not indicate suspicion of csPCa (PI-RADS score ≤ 2) and PSA density < 0.20 ng/ml^2^, following established clinical guidelines [11], patients did not undergo subsequent biopsy and were advised to undergo standard clinical follow-up. This typically involved PSA surveillance.

Histopathological analysis of all biopsy samples was conducted by specialized in-house genitourinary pathologists. The histopathology results were compared to the MRI assessments of both readers according to four prostate quadrants (anterior right/left, posterior right/left) as previously defined for the MRI readout. A lesion with a Gleason score of ≥ 3 + 3 (ISUP/WHO grade group ≥ 1) was categorized as "prostate cancer" (PCa) and a lesion with a Gleason score of ≥ 3 + 4 (ISUP/WHO grade group ≥ 2) was categorized as "clinically significant prostate cancer" (csPCa) [11]. The histopathological biopsy results served as the reference standard to define a PCa/csPCa, while the absence of PCa/csPCa was determined by either a negative biopsy result or a non-suspicious clinical mpMRI result in conjunction with clinical assessment (including comprehensive medical history, PSAD and prostate cancer risk calculators [16], [17]). Patients who did not attend the post-MRI clinical reassessment or/and who did not obtain a biopsy in case of PI-RADS score ≥ 3 were excluded from the study analysis to ensure data consistency.

### 2.5 Statistical analysis

Weighted Cohen’s Kappa (κ) with 95 % confidence intervals (95 %-CI) [18] was calculated to evaluate inter-reader agreement. Interpretation of agreement levels was conducted according to the classification system by Landis and Koch [19].

The clinical standard, based on clinical data and histopathologic results of clinically indicated subsequent prostate biopsies as specified above, served as reference standard to assess the diagnostic performance of ultra-fast bpMRI and mpMRI in detecting csPCa and PCa within the study readout. Accordingly, measures such as sensitivity, specificity, positive predictive value (PPV), and negative predictive value (NPV) with 95 %-CI of ultra-fast bpMRI and mpMRI for the two readers were calculated. To assess the diagnostic performance of the ultra-fast bpMRI protocol in comparison to the mpMRI protocol McNemar’s test (two-tailed) was used due to the paired nature of the study data.

The PI-RADS cutoff values of ≥ 3 and ≥ 4 were established to evaluate the alteration in diagnostic performance in relation to the histopathologic results, encompassing all suspicious lesions (PI-RADS ≥3) and exclusively highly suspicious lesions (PI-RADS ≥4).

To compare the average image quality ratings of the two readers for the ultra-fast bpMRI protocol against the mpMRI protocol, a Wilcoxon signed-rank test (two-tailed) was used.

The level of significance was set to 5 %. The software R (version 4.3.2) [20] was used for all statistical analysis.

## 3 Results

### 3.1 Patient characteristics

Between June 2023 and February 2024, a total of 141 consecutive patients without a previous history of prostate cancer initially fulfilled the inclusion criteria for study participation. However, 17 patients had to be excluded from the final analysis due to the absence of subsequent biopsy despite having a PI-RADS score ≥ 3. Reasons for biopsy omission included personal preference, contraindications against biopsy (such as the need for anticoagulation without cessation), or a low overall clinical suspicion for clinically csPCa, which was determined by considering additional clinical information (e.g., PI-RADS 3 in combination with PSA density < 0.10 ng/ml^2^) [11]. The final study cohort therefore consisted of 123 men, with 62 patients (50.4 %) undergoing subsequent clinically indicated prostate biopsy.

Median age was 64 years (IQR: 58–69 years), and median pre-biopsy PSA-value was 5.4 ng/ml (IQR: 3.6–8.9 ng/ml). Histopathologic biopsy results revealed a PCa (Gleason score ≥ 3 +3, ISUP/WHO grade group ≥ 1) in 44 patients (36.8 %) and a csPCa (Gleason score ≥ 3 +4, ISUP/WHO grade group ≥ 2) in 35 patients (28.5 %).

### 3.2 Inter-reader agreement and diagnostic performance

Inter-reader agreement on PI-RADS scores (see Table 2) was good for both mpMRI (κ = 0.83) and ultra-fast bpMRI (κ = 0.87), indicating a high level of consistency between the readers' evaluations.

**Table 2:** Distribution of assigned PI-RADS scores of both readers evaluating the mpMRI and ultra-fast bpMRI in a cohort of123 patients.

**PI-RADS score**	**mpMRI (n = 123)**		**ultra-fast bpMRI (n = 123)**	
	**Reader 1**	**Reader 2**	**Reader 1**	**Reader 2**
2	66 (53.7 %)	46 (37.4 %)	69 (56.1 %)	53 (43.1 %)
3	5 (4.1 %)	27 (22.0 %)	8 (6.5 %)	23 (18.7 %)
4	30 (24.4 %)	30 (24.4 %)	25 (20.3 %)	26 (21.1 %)
5	22 (17.9 %)	20 (16.3 %)	21 (17.1 %)	21 (17.1 %)

Statistical results of the diagnostic performance of the two readers for both mpMRI and ultra-fast bpMRI protocols are provided in Table 3**.** PI-RADS cutoff values for malignancy of ≥ 3 and ≥ 4 were analyzed in relation to the clinical standard, based on clinical data and histopathologic results of clinically indicated subsequent targeted and systematic biopsies for identifying csPCa based on prostate quadrants.

**Table 3:** Diagnostic performance of the two readers for detecting clinically significant prostate cancer using both mpMRI and ultra-fast bpMRI protocols in a cohort of123 patients.

PI-RADS cutoff	Protocol	Reader	Sensitivity (95 %-CI)	Specificity (95 %-CI)	PPV (95 %-CI)	NPV (95 %-CI)	Accuracy (95 %-CI)
≥ 3	mpMRI	1	94 % (81 %, 99 %)	77 % (67 %, 86 %)	62 % (48 %, 75 %)	97 % (90 %, 1.00)	82 % (74 %, 88 %)
		2	94 % (81 %, 99 %)	53 % (42 %, 64 %)	45 % (33 %, 57 %)	96 % (88 %, 1.00)	65 % (56 %, 73 %)
	ultra-fast bpMRI	1	94 % (81 %, 99 %)	78 % (68 %, 86 %)	63 % (49 %, 76 %)	97 % (90 %, 1.00)	83 % (75 %, 89 %)
		2	94 % (81 %, 99 %)	61 % (50 %, 71 %)	49 % (37 %, 62 %)	96 % (88 %, 1.00)	71 % (62 %, 79 %)
≥ 4	mpMRI	1	94 % (81 %, 99 %)	83 % (73 %, 90 %)	69 % (54 %, 81 %)	97 % (91 %, 1.00)	86 % (79 %, 92 %)
		2	89 % (73 %, 97 %)	81 % (71 %, 88 %)	65 % (49 %, 78 %)	95 % (87 %, 99 %)	83 % (75 %, 89 %)
	ultra-fast bpMRI	1	91 % (77 %, 98 %)	86 % (77 %, 93 %)	73 % (57 %, 85 %)	96 % (89 %, 99 %)	88 % (81 %, 93 %)
		2	91 % (77 %, 98 %)	86 % (77 %, 93 %)	73 % (57 %, 85 %)	96 % (89 %, 99 %)	88 % (81 %, 93 %)

PI-RADS cutoff values for malignancy of ≥ 3 (all suspicious lesions) and ≥ 4 (exclusively highly suspicious lesions) were analyzed in relation to the clinical standard, based on clinical data and histopathologic results of clinically indicated subsequent targeted and systematic biopsies for identifying clinically significant prostate cancer (Gleason score of ≥ 3 + 4, ISUP/WHO grade group ≥ 2).

Reader 1: experienced reader. Reader 2: less experienced reader. PPV: positive predictive value, NPV: negative predictive value, 95 %-CI: 95 % confidence interval.

For PI-RADS cutoff values of ≥ 3, both readers demonstrated high sensitivity (94 %/94 % vs. 94 %/94 %, mpMRI: Reader 1/Reader 2 and ultra-fast bpMRI: Reader 1/Reader 2) and NPV (97 %/97 % and 96 %/96 %) for both the mpMRI and ultra-fast bpMRI protocol. Reader 1 consistently showed higher specificity (77 %/53 % and 78 %/61 %), PPV (62 %/45 % and 63 %/49 %) and diagnostic accuracy (correctly classified proportion, 82 %/65 % and 83 %/71 %) compared to Reader 2, who exhibited a higher rate of false positives. The two readers identified a comparable number of PI-RADS 3 lesions for both protocols respectively; however, Reader 1 had notably fewer PI-RADS 3 lesions compared to Reader 2 (4.1 %/22.0 % and 6.5 %/18.7 %, see Table 2).

For PI-RADS cutoff values of ≥ 4, both readers maintained high sensitivity (94 %/89 % and 91 %/91 %) and NPV (97 %/95 % and 96 %/96 %), but Reader 1 continued to demonstrate mostly higher specificity (83 %/81 % and 86 %/86 %), PPV (69 %/65 % and 73 %/73 %), and diagnostic accuracy (86 %/83 % and 88 %/88 %) compared to Reader 2.

There was no significant difference in the diagnostic performance of correctly identifying csPCa by MRI (PI-RADS cutoff-score ≥3) between the mpMRI protocol and the ultra-fast bpMRI protocol for both readers (p > 0.05).

In an additional analysis, the diagnostic performance of the two readers for detecting prostate cancer (insignificant and significant lesions; Gleason score of ≥ 3 + 3, ISUP/WHO grade group ≥ 1) using both protocols was evaluated (see supplementary Table S1**)** using a PI-RADS cutoff value for malignancy of ≥ 3. In this scenario, both readers maintained high sensitivity (91 %/91 % and 91 %/91 %) and NPV (94 %/92 % and 94 %/93 %). However, Reader 1 again consistently exhibited higher specificity (84 %/57 % and 85 %/66 %), PPV (75 %/54 % and 77 %/60 %), and overall diagnostic accuracy (86 %/69 % and 87 %/75 %) compared to Reader 2.

### 3.3 Image quality analysis

**Fig. 1:**
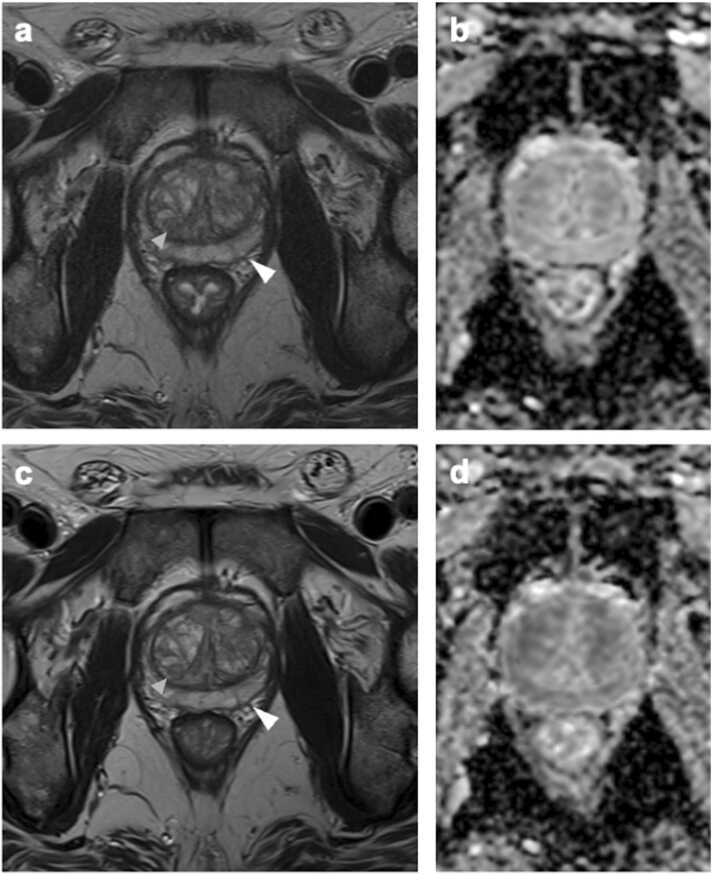
Example for visual comparison of conventional mpMRI (a: T2 TSE, b: DWI) to ultra-fast bpMRI (c: T2 TSE, d: DWI). mpMRI: Slight blurring around prostate capsule (white arrowhead in a) and capsule of hyperplastic nodule in the transition zone (grey arrowhead in a). Acquisition time of full standard protocol: 20 mins 33 sec. ultra-fast bpMRI: Clearer delineation of organ capsule (white arrowhead in c) and capsule of hyperplastic nodule in transition zone (grey arrowhead in c). Acquisition time of full ultra-fast bpMRI: 3 min 28 sec (time savings of approximately −80 %).

The mean mPI-QUAL score by Reader 1 for the mpMRI protocol was 3.5 (SD 0.6) and 3.6 (SD 0.6) for the ultra-fast bpMRI protocol. The mean mPI-QUAL score by Reader 2 for the mpMRI protocol was 3.6 (SD 0.5) and 3.9 (SD 0.4) for the ultra-fast bpMRI protocol. The average image quality ratings of the two readers were significantly better for the ultra-fast bpMRI protocol compared to the mpMRI protocol (p < 0.001). Fig. 1 depicts image examples for visual comparison of both MRI protocols.

## 4 Discussion

MRI has emerged as a pivotal tool in the clinical evaluation of prostate cancer and is now part of all major guidelines with mpMRI being the standard approach advised by the PI-RADS guidelines [11], [21]. However, mpMRI is often time-consuming and costly, restricting its availability. This study aimed to investigate an ultra-fast bpMRI protocol as an alternative to conventional mpMRI with focus on the evaluation of its feasibility and the diagnostic performance in identifying csPCa.

Our results demonstrated a good inter-reader agreement on PI-RADS scores between an experienced and less experienced reader. While both readers showed high sensitivity and NPV for both the ultra-fast bpMRI and full protocol, the more experienced reader mostly had a higher specificity, PPV and overall diagnostic accuracy. The high sensitivity across both readers and protocols suggests that both methods are effective in correctly identifying clinically significant prostate cancer. However, the comparably higher performance metrics for specificity, PPV, and overall diagnostic accuracy as well as the comparably lower absolute number of PI-RADS 3 lesions for Reader 1, suggest that reader experience plays a role especially in reducing indeterminate and even false positive lesions, making the diagnosis more reliable [22]. This aligns with a trial by Kang et al. [23], which also found higher diagnostic performance among more experienced prostate mpMRI readers. It implies that adequate training in reading prostate MRIs is crucial for improving diagnostic accuracy, particularly in interpreting complex cases where the differentiation between clinically significant and insignificant lesions might be subtle [22].

Several studies have shown that bpMRI protocols (without DL-acceleration) offer comparable diagnostic performance compared to the conventional mpMRI protocols [24], [25], [26]. Recently, Oerther et al. [13] found that a DL-accelerated bpMRI protocol demonstrated similar diagnostic performance compared to the conventional mpMRI protocol of the prostate. This is in line with our study results. However, in their study only the mpMRI served as the gold standard. By contrary, our study’s reference standard is derived from the clinical standard, which includes clinical data and histopathology results of clinically indicated subsequent prostate biopsies on a per-quadrant basis.

Another notable finding of our study was that the average image quality was rated slightly, but significantly better for the ultra-fast bpMRI protocol compared to the mpMRI protocol. Gassenmaier et al. [6] and Bischoff et. al. [27], [28] investigated compressed sensing–acquisition and DL–assisted reconstruction methods for T2w imaging of the prostate. They found similar or even improved diagnostic performance with significantly higher image quality using the new reconstruction techniques compared to standard T2w imaging of the prostate. Lee et al. [29], Johnson et al. [30] and Oerther et al. [13] also assed the influence of DL-based reconstruction on the image quality of both T2w and DWI prostate MRI in smaller cohorts (< 80 patients), and also showed similar or even improved image quality for both sequences compared to the conventional protocol. The improved image quality is likely attributable to the reconstruction capabilities of the DL-algorithm [13] and the accelerated acquisition time, which minimizes motion artifacts [31]. Besides reducing motion artifacts, a shorter scan time offers another significant benefit: increased patient comfort and compliance [13]. This is particularly valuable for elderly patients, who often have difficulties to remain still during the MRI examination [32].

Though our study features one of the largest study cohorts to investigate DL-accelerated prostate MRI protocols, the single-center design may limit the generalizability of our findings. Additionally, the fact that not all patients in the study cohort were biopsied (PI-RADS score ≤ 2 and PSA density < 0.15 ng/ml²), and the exclusion of some patients with a PI-RADS ≥ 3 who did not undergo biopsy, could introduce bias. However, this potential bias cannot be avoided as this procedure is in line with current guidelines [11]. Future multi-center studies with larger cohorts are needed to validate our findings, fully establish the clinical utility of ultra-fast bpMRI and explore its long-term impact on patient outcomes, healthcare costs and potential additional applications (e.g., screening examinations).

## 5 Conclusion

Ultra-fast bpMRI protocols offer a promising alternative to conventional mpMRI for the diagnosis of clinically significant prostate cancer. This approach allows for significantly reduced scan time while even optimizing image quality. The good inter-reader agreement and comparable diagnostic performance suggest that ultra-fast bpMRI can enhance prostate cancer management by providing a quicker, and patient-friendly imaging option. However, reader experience remained a considerable factor for diagnostic performance in our study.

## Funding

Antonia M. Pausch receives funding from the Holcim Stiftung zur Förderung der wissenschaftlichen Fortbildung.

## ClinicalTrials number

NCT05903781

## Ethical statement

This prospective single-center study (ClinicalTrials number: NCT05903781) was approved by the institutional review board (Cantonal Ethics Commission Zurich). Written informed consent was obtained from each study participant.

## CRediT authorship contribution statement

**Eberli Daniel:** Writing – review & editing, Resources. **Rupp Niels J.:** Writing – review & editing, Resources. **Hötker Andreas M.:** Writing – original draft, Validation, Supervision, Resources, Project administration, Methodology, Formal analysis, Conceptualization. **Pausch Antonia M.:** Software, Resources, Methodology, Investigation, Formal analysis, Data curation, Conceptualization. **Elsner Clara:** Writing – review & editing, Investigation. **Filleböck Vivien:** Writing – review & editing, Resources, Investigation, Data curation.

## Declaration of Competing Interest

The authors declare the following financial interests/personal relationships which may be considered as potential competing interests: Antonia M. Pausch reports financial support was provided by Holcim Foundation for the Promotion of Scientific Further Education. If there are other authors, they declare that they have no known competing financial interests or personal relationships that could have appeared to influence the work reported in this paper.

## Footnotes

Appendix A Supplementary data associated with this article can be found in the online version at doi:10.1016/j.ejro.2025.100635.
