# Supplementary material for: Ultra-fast biparametric MRI in prostate cancer assessment: Diagnostic performance and image quality compared to conventional multiparametric MRI
Source: Eur J Radiol Open. 2025 Jan 21;14:100635. doi: 10.1016/j.ejro.2025.100635 (PMC11791330; doi:10.1016/j.ejro.2025.100635)
Supplement: Supplementary file 1 — Supplementary material [file mmc1.docx]

# **Supplementary material**

Article title: Ultra-Fast Biparametric MRI in Prostate Cancer Assessment: Diagnostic Performance and Image Quality Compared to Conventional Multiparametric MRI

| PI-RADS  cutoff | Protocol | Reader | Sensitivity (95%-CI) | Specificity (95%-CI) | PPV  (95%-CI) | NPV  (95%-CI) | Accuracy (95%-CI) |
| --- | --- | --- | --- | --- | --- | --- | --- |
| ≥ 3 | mpMRI | 1 | 91% (78%, 97%) | 84% (74%, 91%) | 75% (62%, 86%) | 94% (86%, 98%) | 86% (79%, 92%) |
|  |  | 2 | 91% (78%, 97%) | 57% (45%, 68%) | 54% (42%, 66%) | 92% (80%, 98%) | 69% (60%, 77%) |
|  | ultra-fast bpMRI | 1 | 91% (78%, 97%) | 85% (75%, 92%) | 77% (63%, 87%) | 94% (86%, 98%) | 87% (80%, 92%) |
|  |  | 2 | 91% (78%, 97%) | 66% (54%, 76%) | 60% (47%, 72%) | 93% (83%, 98%) | 75% (66%, 82%) |

**Supplementary TABLE S1.** Diagnostic performance of the two readers for detecting prostate cancer using both mpMRI and ultra-fast bpMRI protocols in a cohort of 123 patients.

PI-RADS cutoff values for malignancy of ≥ 3 (all suspicious lesions) was analyzed in relation to the clinical standard, based on clinical data and histopathologic results of clinically indicated subsequent targeted and systematic biopsies for identifying prostate cancer (Gleason score of ≥ 3 + 3, ISUP/WHO grade group ≥ 1).

Reader 1: experienced reader. Reader 2: less experienced reader.
PPV: positive predictive value, NPV: negative predictive value, 95%-CI: 95% confidence interval.
